# Correction: Migratory autolysosome disposal mitigates lysosome damage

**DOI:** 10.1083/jcb.20240319510092026c

**Published:** 2026-10-27

**Authors:** Takami Sho, Ying Li, Haifeng Jiao, Li Yu

Vol. 223, No. 12 | https://doi.org/10.1083/jcb.202403195 | September 30, 2024

After publication, an error was discovered in the legend of Fig. S3 wherein the descriptions of panels C and D had been accidentally swapped. The figure itself remains unchanged, and the corrected legend is provided here. Minor edits have also been made to the original panel descriptions. Added text is shown in bold, and deletions are crossed out.

**Figure S3.**
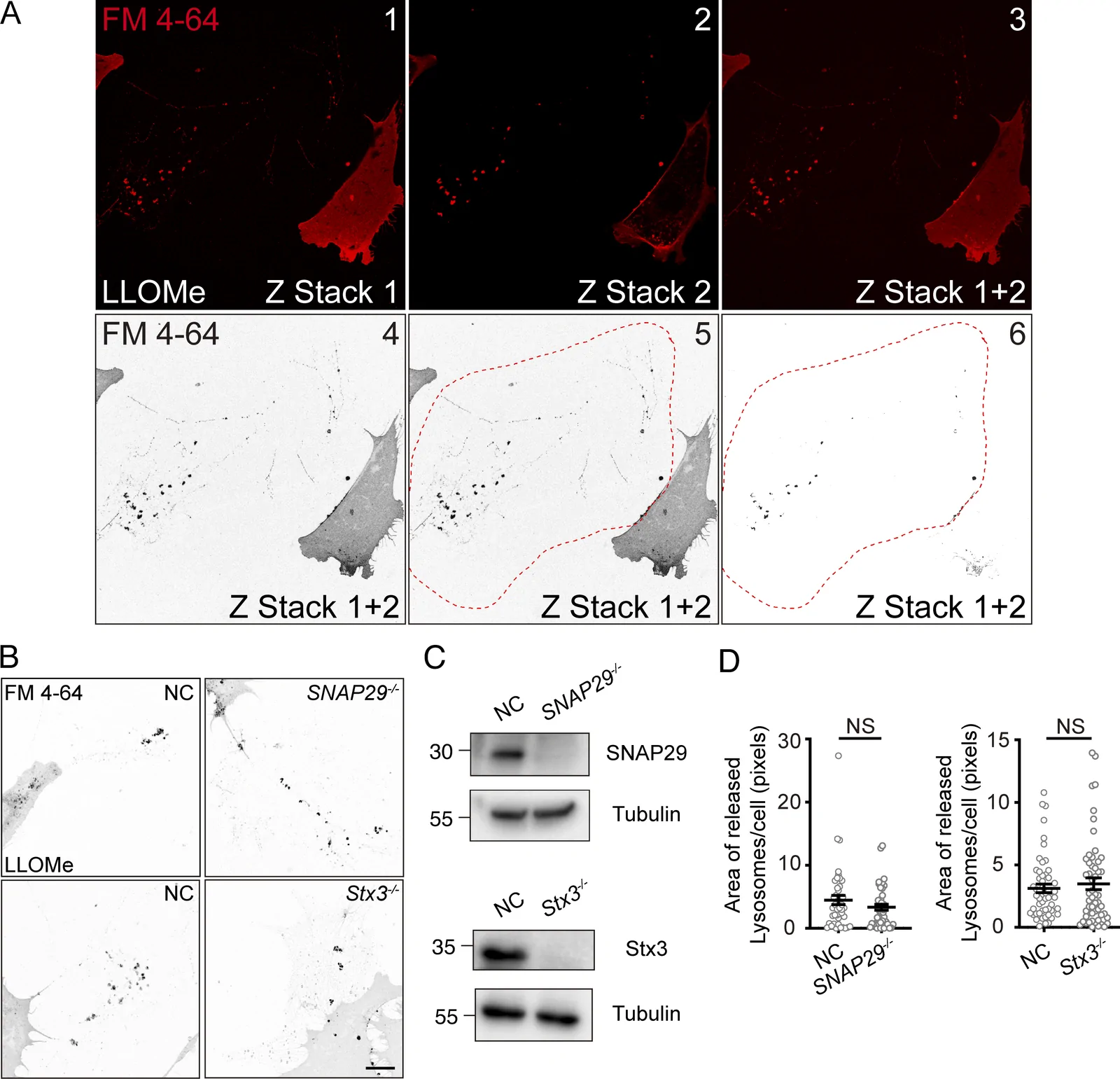
**Related to Fig. 3. (A)** MEF shows the process of image analysis during statistics of extracellular lysosome structures marked with FM 4–64. MEF cells were treated with 2.5 mM LLOMe for 22 h, then fixed with 4% PFA and stained with FM 4–64. Images were captured at two layers (Z = 0.0 μm and Z = 1.0 μm, A1 and A2), then stacked (A3) and converted into gray-value mode (A4). The extracellular region was marked in ImageJ FIJI (A5) and then the threshold was set to the baseline of signal intensity on retraction fibers and plasma membranes (A6). **(B)** Control, *SNAP29*^−/−^, and *Syntaxin3*^−/−^ MEF cells were treated with 2.5 mM LLOMe for 22 h then fixed and stained with FM 4–64. The representative images were converted into gray-value mode. The scale bar denoted 20 μm. **(C)** Western blot results of the samples from experiments in B. Knocking out**Knockout** efficiency of*SNAP29*and*Syntaxin3*was verified respectively was verified for *SNAP29* and *Syntaxin3*, respectively. **(D)**Statistic results**Statistical analysis** of the area of extracellular lysosomal structure of groups in B. Error bars denoted ± SEM from only one fixed-cell imaging experiment; 50 cells were analyzed for all groups. *T* test, NS = not statistically significant.

In the updated panel C description, “Knocking out of *SNAP29* and *Syntaxin3* was verified, respectively” has been changed to “Knockout efficiency was verified for *SNAP29* and *Syntaxin3*, respectively.” In the updated version of panel D, “Statistic results” has been changed to “Statistical analysis” in the sentence “Statistical analysis of the area of extracellular lysosomal structure of groups in B.”

This error does not affect the conclusions of the study. This error appears in print and PDFs downloaded prior to October 22, 2026. The authors apologize for the error and any confusion this may have caused.

